# Pathologic thoracic spine fracture in presence of Parkinson’s disease and diffuse ankylosis: successful management of a challenging condition

**DOI:** 10.1186/1471-2474-14-61

**Published:** 2013-02-11

**Authors:** Yasuchika Aoki, Arata Nakajima, Ryuji Sakakibara, Seiji Ohtori, Kazuhisa Takahashi, Koichi Nakagawa

**Affiliations:** 1 Department of Orthopaedic Surgery, Toho University Sakura Medical Center, 654-1 Shimoshizu, Sakura, Chiba 285-8741, Japan; 2 Department of Internal Medicine, Toho University Sakura Medical Center, Sakura, Chiba, Japan; 3 Department of Orthopaedic Surgery, Graduate School of Medicine, Chiba University, Chiba, Japan

**Keywords:** Parkinson’s disease, Spinal fracture, Ankylosed spine, Ankylosis, Fusion surgery

## Abstract

**Background:**

Patients with Parkinson’s disease have higher risk of complications and revision surgery following spine surgery. Spinal fracture in an ankylosed spine is also difficult to treat. We recently treated a case of thoracic spine fracture in a patient with Parkinson’s disease complicating a severely ankylosed spine. There is no report describing surgical treatment of spine fracture in such a difficult condition, thus, we firstly report the case and discuss the reasons for a successful result.

**Case presentations:**

A 68-year-old man with Parkinson’s disease had a pathologic thoracic spine fracture at T11. Four days after onset, he was referred to a local hospital because of gradually increasing back pain, but no spinal fracture was pointed out at that time. Because he developed lower extremity bilateral numbness and weakness, he was transported to our hospital, eight days after onset. When referred to our hospital, he exhibited severe back pain and paralysis of the lower extremities due to spinal cord involvement. Emergency surgery was performed. Decompression of T10-11 was performed followed by instrumented spinal fusion from T8 to L2. A dramatic neurological improvement was observed following surgery, and complete bony fusion was achieved. At the final two-year postoperative follow-up, the patient had no pathological symptoms related to spinal fracture and no instrument failure was observed.

**Conclusion:**

This patient had Parkinson’s disease and a severely ankylosed spine, both of which may lead to unsatisfactory surgical results from spinal surgery. Generally, patients with Parkinson’s disease have an increased risk for adjacent segment disease and instrument failure. In this patient, fusion surgery did not change the number of fused segments because operated segments were already ankylosed. Because no stress force exists between adjacent vertebral bodies, a severely ankylosed spine may help prevent screw pullout. Thus, treatment of a spinal fracture in an ankylosed spinal segment is a less adverse condition for patients with Parkinson’s disease. Our experience led us to think that a combination of Parkinson’s disease with severely ankylosed spine does not necessarily suggest unsatisfactory outcomes after surgical treatment of spinal fracture.

## Background

Spinal injury in Parkinson’s disease, which is known for its effects on sensorimotor coordination caused by degeneration of the nigrostriatal dopamine pathway, has been reported by many authors [1,2]. Because neuromuscular disorders produce muscular rigidity, shuffling gait, and stooped posture, patients with this disease are at greater risk of falling. Because patients with Parkinson’s disease also usually have poor bone quality [1], falls produce an increased risk of spinal fracture. When a spinal fracture occurs in Parkinson’s patients, surgical treatment is challenging because of the very high complication rate, particularly related to instrumentation [1-3].

An ankylosed spine is also known to be a pathological condition prone to spinal fracture after even trivial trauma [4-9]. It has been reported that patients with ankylosing spondylitis have four times the risk for fractures during their lifetime, compared to unaffected individuals [10]. Patients with diffuse idiopathic skeletal hyperostosis are also at increased risk for spinal fractures, the fracture mechanism being comparable to that of ankylosing spondylitis [8]. Fractures of the ankylosed spine tend to be unstable because of stress concentration at the fracture site; therefore, surgical treatment is usually required. Unfortunately, a delay in diagnosis often occurs. Westerveld et al. reported that 17.1% of fractures occurring in patients with an ankylosed spine were not diagnosed within 24 hour following trauma [8]. Because the fracture is unstable, spinal cord injury frequently occurs, and a delay in diagnosis of the fracture increases the risk of spinal cord injury.

We recently treated a case of thoracic spine fracture in a patient with Parkinson’s disease complicating a severely ankylosed spine, thus, firstly report the case and discuss the reasons for a successful result. Despite a delayed diagnosis and high-risk conditions for surgery, this patient was successfully treated by surgical intervention and obtained full neurological recovery and complete bony fusion.

## Case presentation

**Figure 1:**
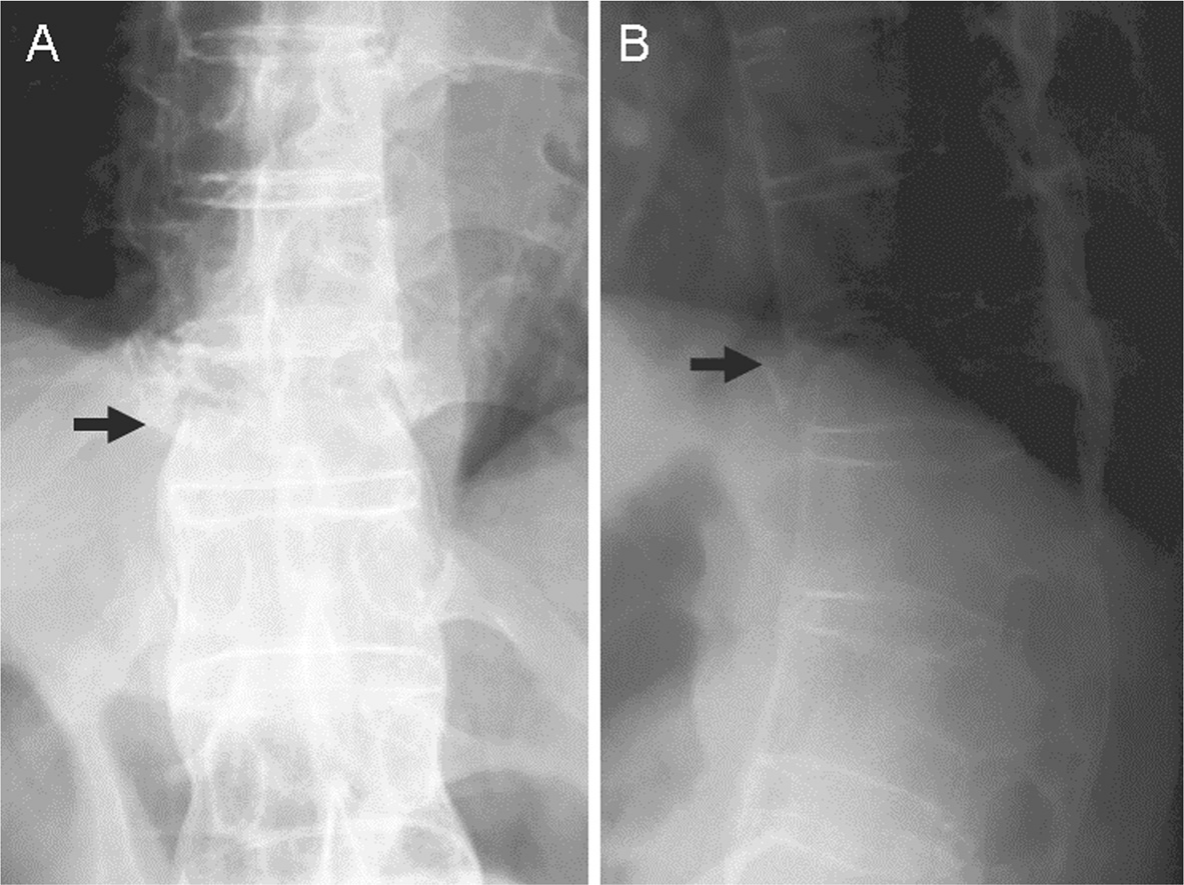
**Anteroposterior (A) and lateral (B) radiographs taken when a patient with Parkinson’s disease was first referred to a local hospital four days after onset of back pain. **Fracture of the T11 vertebral body (arrow) was suspected.

**Figure 2:**
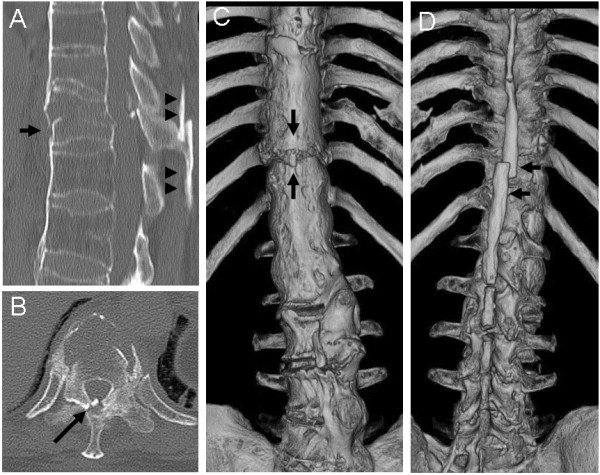
**Computed tomography taken when a patient with Parkinson’s disease was first referred to our hospital eight days after onset of back pain. **The sagittal image (**A**) shows a fracture of the T11 vertebral body (arrow) and displacement of ankylosed spinous processes (arrowheads). The axial image (**B**) shows ossification of the ligamentum flavum at the T10-T11 level (arrow). Three-dimensional images (**C**, **D**) show a severely ankylosed spine at the thoracolumbar levels, and the fracture site is clearly seen (arrows).

**Figure 3:**
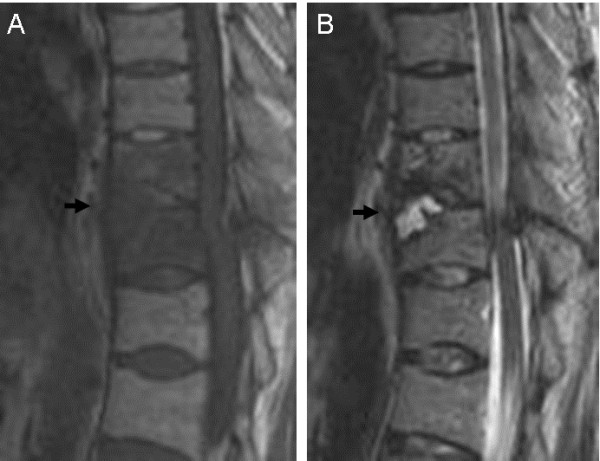
**Preoperative magnetic resonance imaging of a patient with Parkinson’s disease with a thoracic spine fracture in a severely ankylosed spine. **The fracture site (T11: arrow) shows low intensity on the T1-weighted sagittal image (**A**) and high intensity on the T2-weighted sagittal image (**B**), suggesting the presence of fluid accumulation.

A 68-year-old man with Parkinson’s disease and impaired ambulation reported back pain without any traumatic episode. At presentation, he had a 10-year history of Parkinson’s disease. With treatment with 300 mg/day levodopa, his Parkinson’s disease was stage 3 (Hoehn and Yahr), and he could walk without any support, but deteriorated to stage 4 when his medication wore off. Four days after onset, he was referred to a local hospital because of gradually increasing back pain. A radiological examination showed ankylosis of the spine, but no spinal fracture was pointed out at that time (Figure 1). Seven days after onset, he developed lower extremity bilateral numbness and mild weakness. The next day, he was again referred to the hospital, and diagnosed with a thoracic spine fracture. He was then transported to our hospital by ambulance. He reported severe back pain on admission. Physical examination revealed complete motor paralysis and partial sensory loss of the lower extremities (ASIA impairment scale B; ASIA motor score: 50/100). Sensation to light touch and pinprick was intact from C2 to T12, but caudal to T12, sensation to light touch was impaired and sensation to pinprick was absent (ASIA light touch score: 94/112, pin prick score: 76/112). Hyperreflexia was observed in both patellar tendon reflex (PTR) and Achilles tendon reflex (ATR). Computed tomography (CT) showed spinal fracture at the eleventh thoracic (T11) vertebra and the complete ankylosis of the thoracolumbar spine around the fractured site, including the intervertebral discs and spinous processes (Figure 2). CT and magnetic resonance imaging (MRI: Figure 3) revealed the existence of ossification of the ligamentum flavum and spinal cord compression at the T10-T11 level.

**Figure 4:**
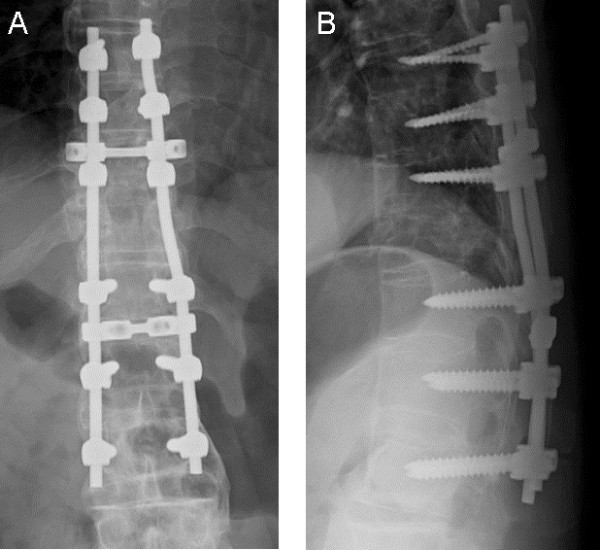
**Anteroposterior (A) and lateral (B) radiographs taken a week after surgery of a patient with Parkinson’s disease with a thoracic spine fracture in a severely ankylosed spine. **Pedicle screws were inserted from T8 to L2 levels, except in the fractured T11 vertebral body.

On the day of admission, we performed posterior decompression and fusion surgery. Laminectomy was performed at the T10-11 levels, and the ossified ligamentum flavum was removed, after which instrumented fusion was performed using pedicle screws (Legacy: Medtronic Sofamor Danek, Memphis, TN) from the eighth thoracic vertebra to the second lumbar vertebra (Figure 4). The resected local bone was applied as a graft bone to the posterior surface of the T10 and T11 lamina. Part of the graft bone was cut into a stick-like shape and put into the gap between the T10 and T11 lamina.

**Figure 5:**
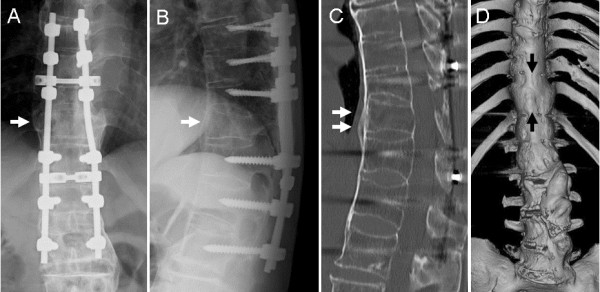
**Anteroposterior (A) and lateral (B) radiographs taken eighteen months after surgery of a patient with Parkinson’s disease with a thoracic spine fracture in a severely ankylosed spine show bony union of the fracture site (T11: arrows) is achieved (arrows) with no evidence of instrument failure. **Sagittal (**C**) and three-dimensional (**D**) images of computed tomography on the same day show more clearly the complete bony fusion (arrows).

A dramatic neurological improvement occurred following the surgery. Two days after surgery, motion of the lower-extremities was first observed (MRC grade 2-3/5 in his quadriceps and hamstrings muscle), and eight days after surgery, he could stand with support (MRC grade 4-5/5 in his quadriceps and hamstrings muscle ASIA motor score: 90/100, light touch score: 112/112, pin prick score: 94/112). Two weeks after surgery, he could walk with a cane wearing a soft thoracolumbar brace, had no back pain, no lower-extremity numbness, and no remaining motor weakness. One month after surgery, physical examination revealed complete recovery of his motor deficit and sensory loss (ASIA impairment scale E, ASIA motor score: 100/100, light touch score: 112/112, pin prick score: 112/112). Four months after the surgery, he could walk without support. Plain radiographs and CT taken eighteen months after the surgery showed the fractured site completely fused with no progression of kyphosis (Figure 5). At the final follow-up two years postoperative, he had no pathological symptoms related to spinal fracture, no progression of kyphosis and no instrument failure. After complete neurological recovery was found one month postoperatively, no motor deficit and sensory loss, except spasticity of lower extremities (hyperreflexia of PTR and ATR), were observed until the final follow-up two years postoperative.

## Conclusions

Surgical treatment of spinal disorder in patients with Parkinson’s disease is often problematic because of its high risk of instrument-related complications and revision surgery [1-3]. Babat et al. reported that revision surgery was required in 12 of 14 of their cases of spine surgery complicated by Parkinson’s disease and concluded that the increased risk is caused by persistent kyphosis or instability at the operated or adjacent vertebral levels [1]. Several reports have also described that patients with Parkinson’s disease frequently have poor bone quality, suggesting increased risk of fractures [11,12]. Despite the higher risk of spinal fracture, there are only a few reports describing the details of surgical results of spinal fracture repair in patients with Parkinson’s disease. In one of the 14 cases reported by Babat et al., a case of L1 osteoporotic burst fracture treated by T11-L2 segmental instrumented fusion was described. Unfortunately, this case required revision surgery because of hook and screw pullout [1]. Nakashima et al. reported three cases of osteoporotic vertebral fracture complicated by Parkinson’s disease, all of which showed significant deterioration of sagittal alignment due to postoperative compression fracture or sinking of the fusion cage, suggesting the difficulty of maintaining sagittal alignment even with posteroanterior surgery [13].

As previously stated, patients with ankylosed spine are prone to spinal fracture, and delay in diagnosis often occurs because of the difficulty of initial diagnosis. Westerveld et al. reported that, of 28 patients with thoracolumbar fracture in patients with ankylosed spine, 27 were the result of hyperextension injury [8]. Our patient had no history of trauma, and showed minimum displacement of fractured site at the first presentation. Ankylosed spine had not been diagnosed before the injury, because kyphosis and spinal rigidity had been thought to be symptoms due to Parkinson’s disease. In such conditions, early diagnosis of spinal fracture in ankylosed spine seemed to be relatively difficult. As reviewed by Heyde et al., beause of pathological tension and shearing force with lacking flexibility due to kyphosis and spinal rigidity, spinal fracture may occur even after minor trauma in ankylosed spine [14]. Secondery osteoporosis and loss of muscle strength also cause the fragility of the spine [14]. Therefore, in severely ankylosed spine, spinal fracture may occur without history of trauma [14,15]. It has been suggested that neurological deficit is due not only to the initial injury, but also to secondary neurological deterioration [9,16]. Because delayed diagnosis of spinal fractures in ankylosed spine often leads to secondary neurological deterioration, clinicians must be cautious not to misdiagnose at first examination even patients have no history of trauma.

Regrettably, the clinical outcome for patients fracturing their ankylosed spine is worse compared to the general spine trauma population, particularly when treated conservatively [8]. Three-column fractures are common in the ankylosed spine and surgical treatment is required in such cases [7-9]. Although Caron et al. reported that 14% of surgically treated patients required revision surgery, no patients required revision surgery when treated by multilevel posterior segmental fixation with at least three bilateral points of fixation above and below the injury [9]. From these observations, long-segment spinal fusion is generally recommended to treat thoracolumbar fracture in the severely ankylosed spine.

Because our patient had a severely ankylosed spine, fusion surgery was performed at levels 3-below and 3-above the fracture level. The patient also had Parkinson’s disease, suggestive of a poor surgical outcome. However, we experienced excellent surgical results with no complications at the two-year follow-up. Because the surgically treated segments had been completely ankylosed before the fracture, it is possible that the excellent results in our case arose from the fact that fusion surgery did not change the number of fused segments. Consequently, the postoperative risk of adjacent segment disease did not increase after fusion surgery in our patient. In addition, the surgically treated levels were completely ankylosed, thus, no stress force existed between adjacent vertebral bodies, except at the fracture site. This fact indicates a decreased risk of pedicle screw breakage or pullout, even though the patient had Parkinson’s disease. A total of six pedicle screws were inserted into the ankylosed vertebral bodies, on cranial and caudal segments respectively, providing more rigidity of pedicle screw fixation. While an ankylosed spine is usually unfavorable for treating patients with spinal fracture, it seems possible that ankylosis around the fracture site may prove advantageous for patients with Parkinson’s disease.

Although our patient had Parkinson’s disease and an ankylosed spine, both of which increase the risk of poor surgical outcomes after spinal surgery, the results for this patient were excellent, suggesting that spinal fracture in an ankylosed spinal segment is a less adverse condition for patients with Parkinson’s disease when treated with long-segment spinal fusion.

To date, there was no previous report describing thoracic spine fracture in a patient with severely ankylosed spine complicated by Parkinson’s disease. However, our experience led us to think that a combination of Parkinson’s disease with severely ankylosed spine does not necessarily suggest unsatisfactory outcomes after surgical treatment of spinal fracture.

## Consent

Written informed consent was obtained from the patient for publication of this Case report and accompanying images. A copy of the written consent is available for review by the Series Editor of this journal.

## Abbreviations

CT: Computed tomography; MRI: Magnetic resonance imaging.

## Competing interests

The authors declare that they have no competing interests.

## Authors’ contributions

Author contributions to the manuscript preparation include the following. YA drafted the manuscript, RS conducted the neurological expertize, KN and AN participated in its design, conception, and acquisition of data, and SO and KT participated in interpretation of data from the viewpoint of spine surgeons. All authors read and approved the final manuscript.

## Pre-publication history

The pre-publication history for this paper can be accessed here:

http://www.biomedcentral.com/1471-2474/14/61/prepub
